# Enhanced Topical Delivery of Tetrandrine by Ethosomes for Treatment of Arthritis

**DOI:** 10.1155/2013/161943

**Published:** 2013-08-24

**Authors:** Chao Fan, Xinru Li, Yanxia Zhou, Yong Zhao, Shujin Ma, Wenjing Li, Yan Liu, Guiling Li

**Affiliations:** ^1^Department of Pharmaceutics, School of Pharmaceutical Sciences, Peking University, Beijing 100191, China; ^2^Institute of Medicinal Biotechnology, Peking Union Medical College and Chinese Academy of Medical Sciences, Beijing 100050, China

## Abstract

The purpose of this work was to explore the feasibility of ethosomes for improving the antiarthritic efficacy of tetrandrine by topical application. It was found that tetrandrine was a weak base (p*K*_*a*_ = 7.06) with pH-dependent partition coefficient. The spherical-shaped ethosomes were prepared by pH gradient loading method. *Ex vivo* permeation and deposition behavior demonstrated that the drug flux across rat skin and deposition of the drug in rat skin for ethosomes was 2.1- and 1.7-fold higher than that of liposomes, respectively. Confocal laser scanning microscopy confirmed that ethosomes could enhance the topical delivery of the drug in terms of depth and quantity compared with liposomes. The ethosomes were shown to generate substantial enhancement of therapeutic efficacy of tetrandrine on Freund's complete adjuvant-induced arthritis with regard to liposomes. These results indicated that ethosomes would be a promising carrier for topical delivery of tetrandrine into and across the skin.

## 1. Introduction

Tetrandrine, a bisbenzylisoquinoline alkaloid extracted from the roots of Stephania tetrandra S. Moore of the Menispermaceae family [1], has many pharmacological effects including anti-inflammatory [2], antioxidation [3], antisilicosis [4], antitumor, antiallergic reaction, and antiplatelet aggregation [5]. It has been therapeutically used for treatment of edema, rheumatic disorders, and inflammatory diseases in clinic [6]. Two dosage forms, tablets and injections, are available, but their applications are limited due to low bioavailability [7], gastric intestine and kidney damage [8], and bad patient compliance [9]. The liver toxicity of tetrandrine in dogs was also found when it was administered chronically [10]. In view of the clinical importance of tetrandrine, the alternative formulations such as aerosol [11] delivered to the lung, nanosphere [12] and hydrogels [13] used in tissue engineering, and solid lipid nanoparticles for intravenous administration [14] have been developed to overcome these drawbacks. Nevertheless, the *in vivo* behavior of these formulations was not evaluated though they exhibited ideal* in vitro *properties. Thus, there still remains a need for tetrandrine to develop a well-proven topical formulation that can solubilize and deliver tetrandrine efficiently and overcome some of the aforementioned drawbacks.

Skin is regarded as the largest organ of human body; its use for topical delivery of drugs has been well documented. Topical drug delivery systems, from a pharmaceutical point of view, offer advantages compared with other routes of administration, including avoidance of first-pass metabolism, fewer administration frequency, smaller fluctuations in plasma drug profile, and good patient compliance [15]. Nevertheless, most of drugs are not suitable for this mode of administration. The main problem in delivery of drugs across the skin is the barrier function of the stratum corneum (SC), which is located in the outermost layer of the skin [16] and consists of corneocytes surrounded by lipid layers. In order to improve topical drug delivery, many kinds of techniques, including complex physical enhancement strategies, such as iontophoresis [17], sonophoresis [18], microneedle [19], and electroporation [20], and lipid vesicular systems such as emulsions [21], microemulsions [22], and liposomal-based delivery systems [23, 24], have been used to overcome the barrier of SC. Amongst these, liposomal-based delivery systems, including conventional liposomes [25], ultraflexible liposomes [26], and ethosomes [24, 27, 28], offer a promising strategy for improving skin drug delivery and have attracted much interest in recent years due to their prominent advantages, including convenience for use and harmlessness to skin. In particular, ethosomes have recently gained attention. Ethosomes were developed by Touitou et al. as additional novel lipid carriers composed of ethanol, phospholipid, and water [24]. Several studies over the past decade have extensively documented the advantages of ethosomes as carriers for topical delivery of drugs over conventional liposomes and ultraflexible liposomes, such as their higher abilities to enhance permeation compared with conventional liposomes [29], and their better compatibility with barriers compared with ultraflexible liposomes containing bilayer fluidizing agent such as sodium cholate [26, 30]. However, there is no document on ethosomal systems for tetrandrine delivery up to date. Consequently, these prompted us to overcome the main complications of available dosage forms for tetrandrine by using ethosomal technique.

In the present study, an ethosomal formulation was developed for topical delivery of tetrandrine through skin. The physicochemical properties of the ethosomes were characterized by the morphology, size and size distribution, and encapsulation efficiency. The *in vitro* skin permeation and deposition behavior of tetrandrine loaded in the ethosomes were assessed by Franz diffusion cells and confocal laser scanning microscopy. In addition, the drug levels in rat plasma following topical application of tetrandrine-loaded ethosomes were also determined in a hope that side effects would be reduced. The pharmacodynamics of the developed tetrandrine-loaded ethosomes in treatment of adjuvant-induced arthritis in rats was evaluated to assess the potential of ethosomes for topical delivery of tetrandrine.

## 2. Materials and Methods

### 2.1. Materials

Tetrandrine was provided by Xian Shanchuan Biotechnology Co., Ltd. (Xian, China). Lipoid S 100 (Phosphatidylcholine, PC) was purchased from Shanghai Dongshang Industries Ltd. (Shanghai, China). Cholesterol (Chol) and Freund's complete adjuvant (FCA) were obtained from Sigma (Shanghai, China). All other chemicals and reagents were of analytical grade or better.

Healthy male Wistar rats weighing 200 ± 10 g and Sprague-Dawley rats (SD rats) weighing 200 ± 20 g were supplied by Animals Center of Peking University Health Science Center. All animals were provided with standard food and water ad libitum and exposed to alternating 12 h periods of light and darkness. Temperature and relative humidity were maintained at 21-22°C and 50%, respectively. All care and handling of animals were performed with the approval of Institutional Authority for Laboratory Animal Care of Peking University.

### 2.2. Determination of Physicochemical Parameters for Tetrandrine

#### 2.2.1. Ionization Constant

The ionization constant *K*_*a*_ of tetrandrine was determined by using nonlogarithmic titration method [31, 32]. In brief, the solutions of tetrandrine with concentrations of 0.5, 1, and 1.5 mmol/L in different concentrations (v/v) of ethanol-water (hydroethanolic) solutions were titrated with 0.01 mol/L hydrochloric acid at room temperature. The titration volume of hydrochloric acid and the pH value were recorded. The p*K*_*a*_ value of tetrandrine was then calculated at different hydroethanolic concentrations.

#### 2.2.2. Partition Coefficient

The octanol/water partition coefficient (*P*) of tetrandrine was determined by using widely applied shake-flask method as our previous description [33]. Briefly, the mixture of *n*-octanol and phosphate-buffered saline (PBS) with different pH value was shaken for 12 h to mutually saturate on a mechanical shaker, and then the *n*-octanol and aqueous phases were separated. Stock solutions of tetrandrine were made in PBS or *n*-octanol, and then merged with equal volume of the two solutions (all were pre-saturated with the other phase). The phases of the solvent system were mutually saturated by shaking for 24 h at room temperature. After centrifugation at 4000 rpm for 30 min, the aqueous phase was carefully taken using a syringe, and the content of tetrandrine in aqueous phase was then determined by HPLC method as described next. The apparent partition coefficient *P*_app_ was calculated. All experiments were carried out in triplicate.

### 2.3. Preparation of Various Tetrandrine Formulations

#### 2.3.1. Preparation of Drug-Loaded Ethosomes

Tetrandrine was encapsulated into ethosomes by using the transmembrane pH gradient loading method as previously reported [34]. Briefly, 200 mg of PC was dissolved in a small volume of ethanol and propylene glycol (1 : 1, v/v) mixture, followed by slower addition of the citrate buffer (pH 3.0) in dropwise under stirring at 700 rpm at 30 ± 1°C. After stirring for additional 5 min and cooling at room temperature, the drug-free ethosomes suspension was obtained. 15.4 mg of tetrandrine (the mass ratio of tetrandrine to PC was kept to be 1 : 13 in each ethosomal system) was immediately mixed with the resultant drug-free ethosomes suspension under stirring at 700 rpm in a closed container. 0.5 mol/L NaOH aqueous solution was then added into the system to adjust the external pH of ethosomes to 6.9–7.1, followed by incubation at 40°C for 1 h. The unionized drug could cross the lipid bilayer and was entrapped into the vesicles. Then, the resultant suspension was extruded 5 times through polycarbonate filters with 100 nm pore size to obtain homogeneous tetrandrine-loaded ethosomes. The ethosomal system with 1.5 mg/mL, 1.0 mg/mL, and 0.3 mg/mL of tetrandrine, respectively, was obtained.

The coumarin-6-loaded ethosomes containing 0.0002% coumarin-6, 2% PC, and 45% ethanol-propylene glycol mixture (1 : 1, v/v) were prepared by the classic passive loading method as elsewhere reported [24]. PC and coumarin-6 were dissolved in a small volume of ethanol-propylene glycol mixture. PBS (pH 7.4) was added slowly to the resultant solution in dropwise with continuous stirring at 700 rpm in a closed container at 30 ± 1°C. Mixing was continued for additional 5 min and then left to cool at room temperature. At last, the preparation was extruded through polycarbonate membrane (100 nm) 5 times to get fluorescent ethosomes.

#### 2.3.2. Preparation of Drug-Loaded Liposomes

Tetrandrine-loaded liposomes were prepared by the thin film hydration method [25, 28, 35, 36], followed by the sequential extrusion. Briefly, PC and Chol (10 : 1, w/w) and tetrandrine were dissolved in chloroform in a round-bottom flask and then evaporated under vacuum at room temperature to form a thin and homogeneous film. The resulted dry films on the inside wall of round bottom flask were hydrated and dispersed with citrate buffer (pH 7.0) at room temperature. Then the suspension was extruded 5 times through polycarbonate filters with 100 nm pore size to obtain homogeneous tetrandrine-loaded conventional liposomes. The coumarin-6-loaded liposomes were prepared as described previously.

#### 2.3.3. Preparation of Tetrandrine Solution

15 mg of tetrandrine was dissolved in 10 mL of 45% ethanol/propylene glycol (1 : 1, v/v) solution in water to obtain 1.5 mg/mL tetrandrine solution, which was used as a control.

### 2.4. Physicochemical Characterization of Tetrandrine-Loaded Vesicles

#### 2.4.1. Particle Size and Size Distribution

The particle size and polydispersity index (PDI) of tetrandrine-loaded vesicles were determined by dynamic light scattering (DLS) (Zetasizer ZEN 3600, Malvern, UK). All DLS measurements were performed with a scattering angle of  90° at 25°C after diluting the dispersion to an appropriate volume with citrate buffer (pH 7.0). The results were the mean values of three samples.

#### 2.4.2. Transmission Electron Microscope

The morphology of tetrandrine-loaded ethosomes was visualized by transmission electron microscope (TEM, JEM-1230, JEOL, Japan). A droplet of ethosomes suspension diluted with citrate buffer (pH 7.0) was allowed to settle on a copper grid with carbon film for minutes, followed by removal of the excess fluid with filter paper. Then a drop of  1 wt% uranyl acetate was placed on the above grid and kept for minutes, and the residual solution was removed by filter paper. The grid was dried for 48 h before examination on TEM at an acceleration voltage of 80 kV.

#### 2.4.3. Encapsulation Efficiency

The encapsulation efficiency (EE) of vesicles was measured by using the ultrafiltration method. The EE was calculated as the percentage of the loaded tetrandrine over the original feeding amount of tetrandrine as described below. In brief, the tetrandrine-loaded vesicles were ultrafiltered with a filtration membrane with molecular weight cutoff of 30 kD (Milipore Co. Ltd., USA), and the content of tetrandrine in the ultrafiltrate was measured by HPLC method as described next. The EE was calculated as follows:

(1) EE(%)=the feeding amount of tetrandrine−the amount of tetrandrine in the ultrafiltratethe feeding amount of tetrandrine×100%.

Tetrandrine was quantified in ultrafiltrates on a Shimadzu series HPLC system (Shimadzu LC-10AT, Kyoto, Japan) equipped with a UV detector (Shimadzu SPD-10A) and reversed phase column (ODS C18, 5 *μ*m, 4.6 mm × 250 mm, China). The mobile phase consisted of acetonitrile/water/acetic acid (70/30/1, v/v) with 0.1% (w/v) sodium dodecyl sulfonate and was pumped at a flow rate of 1.0 mL/min. The detection wavelength was 232 nm. The column temperature was set at 25°C.

### 2.5. *Ex Vivo* Skin Penetration and Deposition Studies

Normal SD rat skin was used as the barrier membrane. The *ex vivo* skin penetration and deposition experiments of different tetrandrine formulations were run in Franz vertical diffusion cells with an effective penetration area and receptor cell volume of 1.76 cm^2^ and 13 mL, respectively. The dermatomed skin was mounted on a receptor compartment with the stratum corneum side facing upward into the donor compartment. The donor compartment consisted of 2 mL of different tetrandrine-loaded formulations, which was then covered with a parafilm to avoid any evaporation process. The receptor compartment was filled with citrate buffer (pH 3.0) and stirred gently by a magnetic stirrer (84-1A, Shanghai, China). The temperature was maintained at 37 ± 0.5°C throughout experiments, in order to maintain the skin surface at 32 ± 1°C. At predetermined time intervals, 1.0 mL aliquots of the receptor medium were withdrawn and immediately replaced with an equal volume of fresh buffer, followed by filtration and analysis of the concentration of tetrandrine by HPLC as described previously. All experiments were conducted in triplicate. Sink conditions were maintained throughout all the experiments.

The skin was removed from Franz diffusion cells at the end of the permeation experiment and washed three times with warm receptor medium. Then, the skin was cut into small pieces, and the drug in the skin was extracted in 1 mL of methanol under shaking at 37°C for 2 h. The resultant methanol solution was filtered through 0.22 *μ*m polycarbonate membrane and analyzed by HPLC as described previously. The skin deposition of tetrandrine was calculated according to the following equation:

(2) skin deposition of tetrandrine(%)  =the content of drug deposited in the skintotal content of administered drug×100%.

### 2.6. Confocal Laser Scanning Microscopy Observation

Confocal laser scanning microscopy (CLSM) was used to determine depth and mechanism of skin permeation of different formulations. Briefly, the tested formulations (coumarin-6-loaded ethosomes, coumarin-6-loaded liposomes, and coumarin-6 solution) were applied nonocclusively to the dorsal skin of Wistar rats for 10 h, respectively. The rat was then sacrificed, and dorsal skin was excised and cleaned with a thin stream of water to remove any residual formulation; then, the adhering fat and/or subcutaneous tissue was removed. Thereafter, the skin sample was sliced in sections of 10–15 *μ*m thickness through the *z*-axis and examined with CLSM (Leica SP2, Heidelberg, Germany). 

### 2.7. Assessment of *In Vivo* Antiarthritic Activity

Adjuvant-induced arthritis was induced in male Wistar rats as previously described [37]. 56 male Wistar rats were randomly divided into eight equal groups as follows:

1. the normal control group which received no adjuvant-induced arthritis and medication,
2. the negative control group which topically received saline,
3. the positive control group which received the topical market dexamethasone ointment (999, Shenzhen, China),
4. the high dose group which topically received tetrandrine-loaded ethosomes at a high dose of 0.45 mg/rat (i.e., 0.3 mL/rat for tetrandrine-loaded ethosomes with 1.5 mg/mL of tetrandrine),
5. the medium dose group which topically received tetrandrine-loaded ethosomes at a medium dose of 0.3 mg/rat (i.e., 0.3 mL/rat for tetrandrine-loaded ethosomes with 1.0 mg/mL of tetrandrine),
6. the low dose group which topically received tetrandrine-loaded ethosomes at a low dose of 0.09 mg/rat (i.e., 0.3 mL/rat for tetrandrine-loaded ethosomes with 0.3 mg/mL of tetrandrine),
7. the liposomes group which topically received tetrandrine-loaded liposomes at a dose of 0.45 mg/rat (i.e., 0.3 mL/rat for tetrandrine-loaded liposomes with 1.5 mg/mL of tetrandrine),
8. the solution group which topically received tetrandrine solution at a dose of 0.45 mg/rat (i.e., 0.3 mL/rat for tetrandrine solution with 1.5 mg/mL of tetrandrine).

The antiarthritic effect of the tested formulations was simultaneously monitored. Arthritis was induced by subplantar injection of 0.1 mL of FCA into the right posterior paw of each rat except the normal group, 1 h before the first drug administration (day 1) where maximum oedema was reached. The initial paw size was then determined using YLS-7B plethysmometer (Jinan, China). Each group received its medication, and the oedema volume of the right paws was then measured at 18 h and 66 h after the first drug administration using plethysmometer, respectively, to evaluate the antiarthritic activity in an acute inflammation. 

At day 7 after the first drug administration, the rats in each group except the normal group were singly medicated for the left paw and then once daily, which was designed on a prophylactic schedule to assess prevention of deuteropathy, and the oedema volume of the left paw was measured before the drug administration at day 10, 13, 15, and 18. The antiarthritic efficacy of each formulation was evaluated by the degree of hind paw swelling and inhibition rate, which was calculated by the following formula:

(3) $$\begin{array}{c} \text{swelling}\;\left(\%\right)=\frac{V_{t,t}-V_{t,0}}{V_{t,0}}\times 100,\\ \text{inhibition}\;\left(\%\right)=\frac{\left(V_{t,t}-V_{t,0}\right)-\left(V_{n,t}-V_{n,0}\right)}{V_{n,t}-V_{n,0}}\times 100, \end{array}$$

where *V*_*t*,*t*_ and *V*_*t*,0_ are the oedema volumes of each tested group at different time and before the first drug administration, respectively; *V*_*n*,*t*_ and *V*_*n*,0_ are the oedema volumes of the negative control group at different time and before the first administration of normal saline, respectively.

### 2.8. Determination of Tetrandrine Plasma Concentration in Rats

#### 2.8.1. Administrations

15 normal male Wistar rats weighing 200 ± 10 g were randomly divided into three equal groups and fasted overnight but allowed access to water ad libitum prior to the experiments. For one group, a day before the experiment the back area was shaved using an electrical clipper. The tetrandrine-loaded ethosomes formulation was applied on the dorsal skin of each of the five rats at a dose of 0.45 mg/rat. The rats were restricted by hands for 5 min after application and then placed in individual cages. The other two groups were given tetrandrine solution via caudal vein injection at doses of 2.5 mg/kg and 30 mg/kg, respectively.

#### 2.8.2. Sampling and Determination

Blood samples of 0.2 mL were collected from orbital venous plexus of the rats at predetermined time intervals after administration. The blood samples were immediately put into heparinized tubes and then centrifuged at 5000 rpm for 10 min. The plasma was collected and stored at −20°C until HPLC analysis. Liquid-liquid extraction procedures for plasma samples were as follows: 2 *μ*L of internal standard solution (1 mg/mL of evodiamine in methanol) was added into 5 mL polypropylene screw-capped conical tubes and then evaporated under a stream of nitrogen, followed by addition of 100 *μ*L of the plasma and 100 *μ*L acetonitrile. After vigorous vortex mixing for 2 min, the upper organic layer was collected by centrifugation at 13000 rpm for 15 min. The upper organic phase was analyzed by HPLC at the wavelength of 225 nm. 20 *μ*L of aliquot was injected into the HPLC system (Shimadzu LC-10AT, Kyoto, Japan) equipped with a UV detector (Shimadzu SPD-10A) and reversed phase column (ODS C18, 5 *μ*m, 4.6 mm × 250 mm, Agela, China) at 30°C. The mobile phase consisted of acetonitrile/water/acetic acid (50/50/0.8, v/v) with 0.1% (w/v) sodium dodecyl sulfonate and was pumped at a flow rate of 1.0 mL/min. The coefficient of variation of the interday and intraday precision of the quality control samples ranged from 0.84 to 3.90%, and accuracy ranged from 97.5 to 105.6%. The limit of detection and the limit of quantification were 100 ng/mL and 500 ng/mL, respectively. The extraction recovery was higher than 90%.

### 2.9. Statistical Analysis

All data were expressed as mean standard deviation (SD) unless particularly outlined. The statistical significance of differences amongst more than two groups was determined by one-way ANOVA by the software SPSS 13.0. Values of *P* < 0.05, *P* < 0.01, and *P* < 0.001 were considered to be significant, highly significant, and extremely significant, respectively.

## 3. Results

### 3.1. Physicochemical Parameters for Tetrandrine

**Figure 1:**
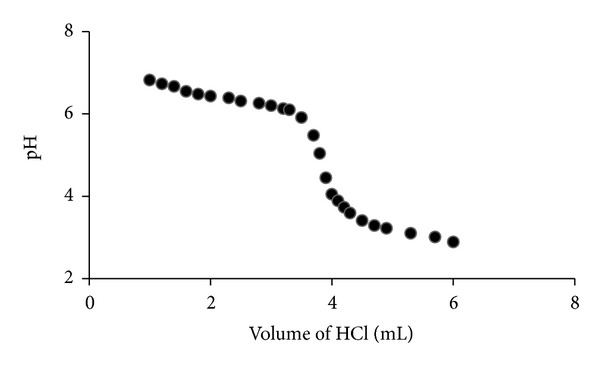
Representative titration curve of tetrandrine in 40% ethanol-water solution.

The aqueous ionization constant (*K*_*a*_) and partition coefficients are very important physicochemical parameters for drugs to be formulated in the pharmaceutical science due to the fact that the existence form of drug molecules could affect their entrapment capacity. Furthermore, ionizable molecules have relatively poor skin permeation due to the diffusional resistance of the lipidic stratum corneum [28]. Taking into account that the alkaloid tetrandrine was encapsulated in liposomes and ethosomes for topical delivery via skin, it was necessary to evaluate these parameters in detail. Since many drugs are poorly water soluble, the *K*_*a*_ value cannot be determined by common methods such as potentiometry, whereas the mixed solvent approach can be exploited. In the present study, the apparent *K*_*a*_ value of tetrandrine was determined in ethanol-water (hydroalcoholic) systems with different concentrations. The representative titration curve of tetrandrine in 40% (v/v, 0.5 mmol/L) hydroalcoholic solution was shown in Figure 1, the apparent p*K*_*a*_ value was calculated to be 6.43. The apparent p*K*_*a*_ values of tetrandrine in other concentrations of hydroalcoholic solutions were presented in Table 1. Based on these results, the mean p*K*_*a*_ value of 7.06 for tetrandrine in water was obtained, suggesting that the pH of the medium must be higher than the p*K*_*a*_ of tetrandrine in order to get higher encapsulation efficiency. However, the pH could not be too high because the lipid bilayer might collapse under high pH condition [34].

**Figure 2:**
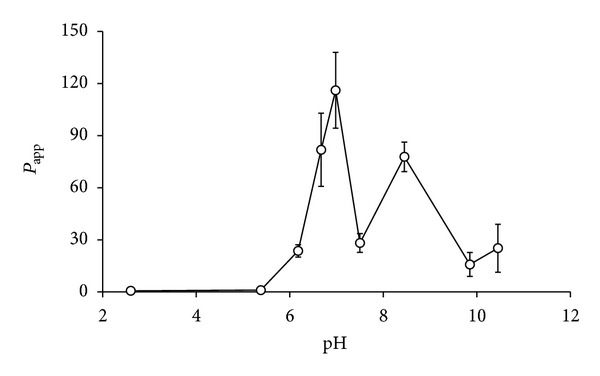
Effect of pH of PBS on apparent partition coefficients of tetrandrine (*n* = 3).

Next, the effect of pH on the apparent partition coefficients *P*_app_ of tetrandrine was assessed. As shown in Figure 2, the *P*_app_ value of tetrandrine was essentially kept constant at pH < 5.38, then increased profoundly up to pH 7.0 and decreased abruptly thereafter and fluctuated in a certain range within pH 6.67–10.45. Overall, tetrandrine exhibited the highest apparent partition coefficient at pH 7.0 for PBS.

Based on these results of p*K*_*a*_ and *P*_app_, it was concluded that tetrandrine was present in molecular form and exhibited the highest hydrophobicity at about pH 7.0. Therefore, pH 7.0 was selected for the subsequent studies.

### 3.2. Characterization of Tetrandrine-Loaded Vesicles

The prepared tetrandrine-loaded ethosomes were characterized by morphology, particle size and size distribution, and entrapment efficiency.

**Figure 3:**
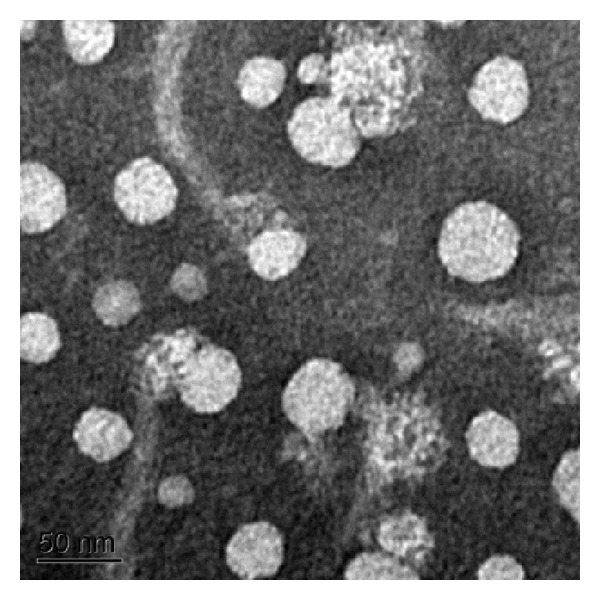
Transmission electron micrograph of tetrandrine-loaded ethosomes.

The examination of the prepared tetrandrine-loaded ethosomes by TEM shown in Figure 3 clearly indicated the predominance of spherical-shaped vesicles, which were discrete, more or less uniform in size, and appeared to be unilamellar.

Particle size and size distribution for vesicles were determined by DLS. The mean diameter of tetrandrine-loaded ethosomes and liposomes was 78.03 ± 0.24 nm (PDI = 0.238 ± 0.012) and 99.86 ± 0.48 nm (PDI = 0.232 ± 0.004), respectively, indicating that the size of ethosomes was considerably smaller than that of liposomes, and both ethosomes and liposomes exhibited a narrow particle size distribution. Noteworthy, the size of ethosomes observed by TEM appeared to be smaller than that determined by DLS, which might be due to the dehydration and collapse of the hydrophilic corona of the vesicles during drying and staining of the TEM specimen [38].

The EE of tetrandrine in ethosomes and liposomes was determined by using the ultrafiltration method. Taken individually, the EE for liposomes was 98.8 ± 0.01% (*n* = 3), whereas for ethosomes 52.87 ± 3.8% (*n* = 3), indicating that the EE of liposomes was almost twice as high as that of ethosomes.

### 3.3. Delivery of Tetrandrine Into/Across the Skin

**Figure 4:**
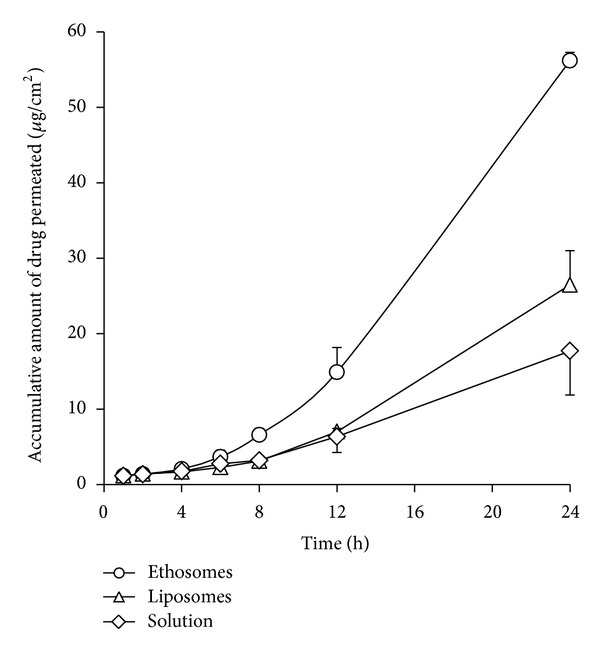
Comparative cumulative amount of tetrandrine permeated from ethosomes, liposomes, and drug solution in a 24 h study via rat skin (*n* = 3).

The skin permeation of tetrandrine from different formulations such as ethosomes, liposomes, and solution into and across rat skin was evaluated in Franz vertical diffusion cells. The cumulative amount of tetrandrine permeated per unit area across excised rat skin via various formulations was plotted as a function of time (Figure 4). The time-dependent permeation curves of tetrandrine showed classic permeation behavior, with a lag phase followed by steady state flux. The value of steady state flux was calculated from the slope of the linear portion in Figure 4. It was found that tetrandrine flux from ethosomes (3.18 ± 0.15 *μ*g/cm^2^/h) was extremely significantly higher than that from liposomes and solution with flux of 1.50 ± 0.32 *μ*g/cm^2^/h and 0.92 ± 0.33 *μ*g/cm^2^/h, respectively (*P* < 0.001). However, there was no significant difference in flux between liposomes and solution (*P* > 0.05).

**Figure 5:**
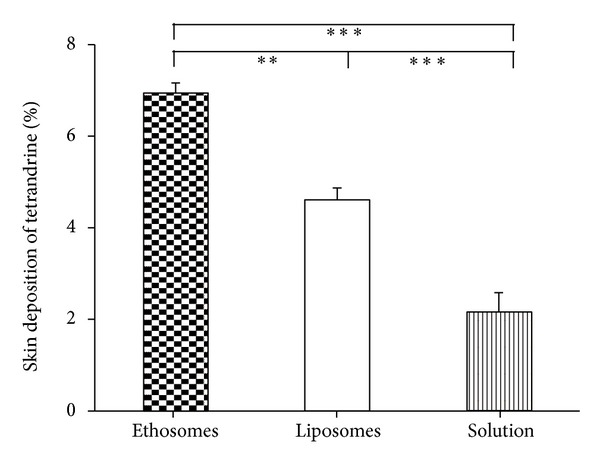
Skin deposition of tetrandrine after 24 h of application of ethosomes, liposomes, and drug solution, respectively (*n* = 3). **P < 0.01, ***P < 0.001.

Skin deposition of the drug at the end of the 24 h experiment for different formulations was shown in Figure 5. Excitedly, ethosomes also facilitated the best skin deposition amongst the three formulations tested here. The deposition of tetrandrine in the skin was extremely significantly higher for ethosomes (6.95 ± 0.22%, w/w) and liposomes (4.10 ± 0.79%, w/w) as compared with solution (2.60 ± 0.44%, w/w) (*P* < 0.001). Highly significant difference in the content of tetrandrine in the skin was also observed between ethosomes and liposomes (*P* < 0.01).

### 3.4. CLSM Observation

**Figure 6:**
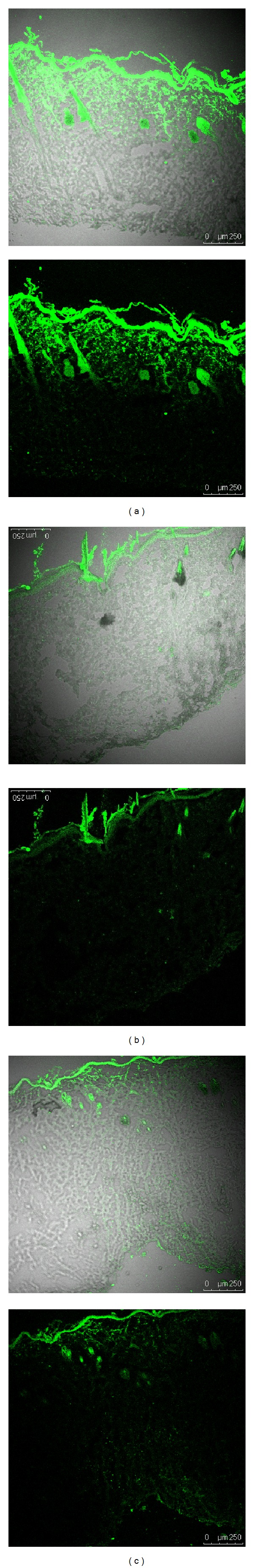
CLSM images of rat skin after 8 h application of the coumarin-6 solution (a), coumarin-6-loaded liposomes (b), and coumarin-6-loaded ethosomes (c), respectively.

CLSM of rat skin after 8 h application of coumarin-6 from solution, liposomes, and ethosomes was shown in Figures 6(a)–6(c), respectively. Using 45% ethanol/propylene glycol (1 : 1,v/v) solution of coumarin-6, the confocal photomicrographs showed that the penetration from coumarin-6 solution possibly by way of hair follicle was confined to the upper layer of the skin, and the penetration depth was up to the last layer (stratum basale) of epidermis but of relatively very low fluorescence intensity (Figure 6(a)). However, the use of liposomes and ethosomes resulted in an obvious decrease in the deposition of coumarin-6 in the upper layer of the skin (Figures 6(b) and 6(c)), indicating that liposomes and ethosomes might not penetrate the skin through hair follicle. Noteworthy, the fluorescence intensity ranging from stratum granulosum to stratum basale from ethosomes was significantly stronger than that from liposomes (Figures 6(c) and 6(b)), whereas there was no remarkable difference in the fluorescence intensity below the stratum corneum between solution and liposomes (Figures 6(a) and 6(b)), indicating that coumarin-6 from ethosomes could easily penetrate into deeper layers of the skin and increase the amount of drug in the skin (Figure 6(c)) and liposomes could not permeate through the stratum corneum barrier. Overall, ethosomes could enhance the delivery of coumarin-6 in terms of depth and quantity compared with liposomes.

### 3.5. *In Vivo* Antiarthritic Efficacy

**Figure 7:**
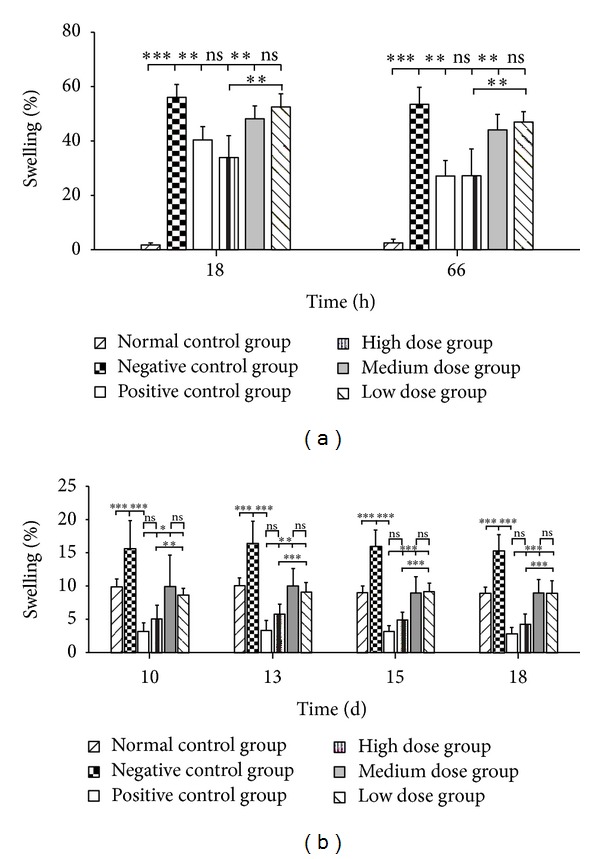
Effect of the dose of topical tetrandrine-loaded ethosomes on inhibition of oedema in rats for primary adjuvant arthritis (a) and secondary adjuvant arthritis (b) (*n* = 7). *P < 0.05; **P < 0.01; ***P < 0.001; ns, *P* > 0.05.

**Figure 8:**
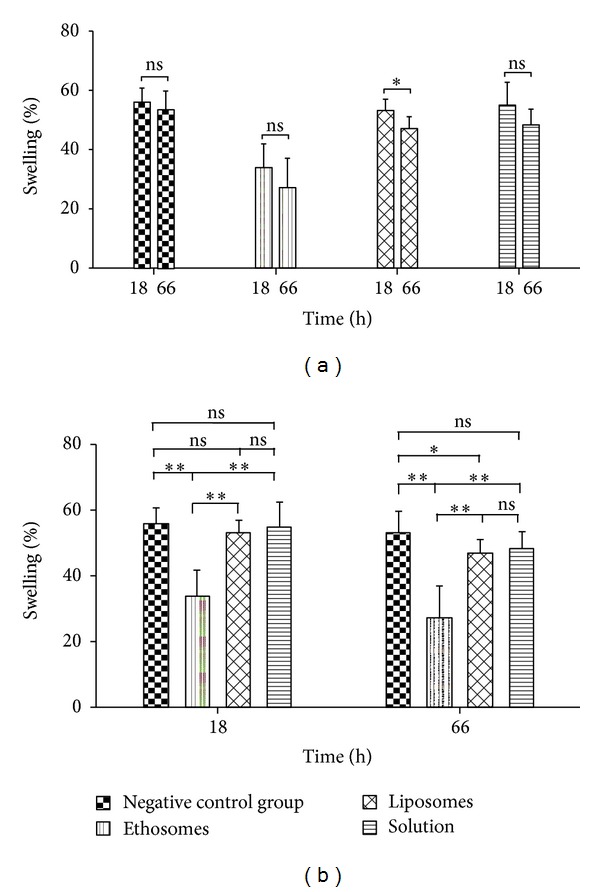
Effect of topical application of tetrandrine formulations at a single dose of 0.45 mg/rat on primary adjuvant arthritis in rats (*n* = 7). *P < 0.05; **P < 0.01; ns, *P* > 0.05.

**Figure 9:**
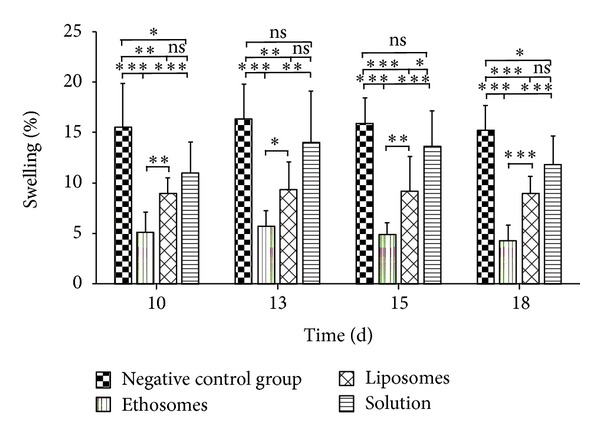
Effect of topical daily application of different tetrandrine formulations on secondary adjuvant arthritis in rats after 7 days (*n* = 7). *P < 0.05; **P < 0.01; ***P < 0.001; ns, *P* > 0.05.

To examine the effect of the topical tetrandrine formulations on primary and secondary adjuvant arthritis of rats, the change in paw oedema volume in rats after topical application of tetrandrine formulations as function of time and treatment was estimated by swelling degree and inhibition rate. Normal saline was also topically administered as negative control and dexamethasone ointment as positive control. Figures 7, 8, and 9 showed the mean swelling degree after topical application of the tested formulations to arthritis-inducing regions of the rat's right hind paw and the rat's left hind paw, respectively. First, as shown in Figure 7(a) for primary adjuvant arthritis, the swelling degree for the negative control group was extremely significantly greater than that for the normal control group (*P* < 0.001), and the swelling degree for the positive control group was highly significantly lower than that for the negative control group (*P* < 0.01). Similar results were also obtained as seen from Figure 7(b) for secondary adjuvant arthritis in rats. These suggested that the animal model of arthritis induced by FCA was successfully set up. 

Further, the effect of the dose of topical tetrandrine-loaded ethosomes on inhibition of paw oedema in rats was evaluated. As shown in Figure 7(a), at 18 h and 66 h after the first drug administration, there was highly significant difference in the swelling degree between the high and medium dose groups (*P* < 0.01), and the swelling degree for the medium dose group was lower than that for the low dose group but with no significant difference (*P* > 0.05). The inhibition rate of oedema in rats was 1.85%, 13.07%, and 36.80% at 18 h, and 7.10%, 16.97%, and 46.48% at 66 h for low, medium, and high dose groups, respectively (Table 2). These indicated that the therapeutic impact of primary arthritic efficacy for tetrandrine-loaded ethosomes was highly dependent on the dose of tetrandrine. Similar trends could be seen from Figure 7(b) and Table 2 for secondary adjuvant arthritis.

Next, the treatment effect of different tetrandrine formulations at high dose on rat's primary adjuvant arthritis was assessed.  Figure 8 summarized the change in the oedema volume in rats as function of time and treatment.  Figure 8(a) and Table 2 showed that the tested tetrandrine formulations reduced the oedema volume in rats with time compared to the negative control group. Notably, for the ethosomes group the therapeutic impact of a single dose was evident over the entire test period of 66 h (Table 2) and was highly significant compared to the negative control group (*P* < 0.01) (Figure 8(b)). Moreover, as seen from Figure 8(b), the oedema volume (% of initial) in rats at 18 h for ethosomes group (33.91%) was highly significantly decreased (*P* < 0.01), for liposomes group (52.23%) and for solution group (55.00%) was not significantly decreased (*P* > 0.05), compared to the negative control group (56.05%). The swelling degree for ethosomes group was highly significantly lower than that for liposomes group and solution group (*P* < 0.01), and there was no significant difference in swelling degree between liposomes group and solution group (*P* > 0.05). Similar results were also observed at 66 h (Figure 8(b)). These indicated that the tetrandrine-loaded ethosomes had the best effect amongst the tested tetrandrine formulations on primary adjuvant arthritis. A demonstration of this best formulation was shown in Table 2 where the inhibition rate was 36.80%, 1.32%, and 1.60% at 18 h, and 46.48%, 7.79%, and 8.63% at 66 h for ethosomes, liposomes, and solution groups, respectively. Additionally, on comparing the antiarthritic efficiency of the tetrandrine-loaded ethosomes formulation to the efficiency of the market dexamethasone ointment at a dose of 0.1 g cream (i.e., 0.1 mg drug/rat), it was found from Figure 7(a) that the two formulations exhibited comparative inhibition of the induced oedema over the entire test period of 66 h (*P* > 0.05); the swelling degree was 33.91% and 40.36% at 18 h, 27.20% and 27.14% at 66 h for the ethosomes and commercial formulation, respectively. Nevertheless, it was worth noting that the ethosomes generated rapid reductions in the oedema volume and exhibited higher inhibition rates compared with the commercial formulation (Figure 7(a) and Table 2).

 Finally, the treatment effect of different tetrandrine formulations on rat's secondary adjuvant arthritis was evaluated.  Figure 9 revealed the maximum increase in oedema volume in rats receiving no treatment (negative control group) after 13 days with mean increase value equivalent to 16.34%, and then a gradual decrease followed reaching 15.23% after 18 days. Compared to the negative control group, there was a highly or extremely significant reduction in paw oedema in all drug-treated groups (*P* < 0.001 for ethosomes, *P* < 0.01 at day 10 and 13 and *P* < 0.001 at day 15 and 18 for liposomes, resp.) except the solution group (*P* > 0.05) at each interval after-treatment, indicating that ethosomes and liposomes formulations had a better effect on secondary adjuvant arthritis in rats compared to solution formulation. In addition, it was found that the paw edema volume in ethosomes group was significantly reduced compared to liposomes group, whereas there was no significant difference between the liposomes group and solution group (*P* > 0.05). These results were essentially consistent with those obtained from the treatment efficacy of primary adjuvant arthritis in rats aforementioned. Furthermore, no significant difference in the paw edema volume (% of initial) in rats between positive control group (market dexamethasone ointment) and ethosomes group (*P* > 0.05) was observed (Figure 7(b)). Nevertheless, the inhibition rates of the market formulation were higher than those of ethosomes (Table 2), which was opposite to the result of primary adjuvant arthritis in rats aforementioned (Table 2). Taken together, ethosomes seemed to be an optimal formulation for topical delivery of tetrandrine.

### 3.6. Tetrandrine Levels in Plasma

**Figure 10:**
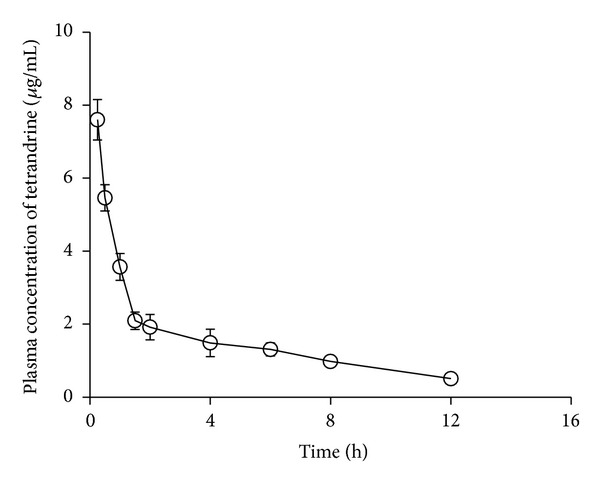
Tetrandrine plasma concentration profile after intravenous injection of tetrandrine solution to rats at a single dose of 30 mg/kg (*n* = 5).

The mean plasma drug concentration profiles following topical administration of tetrandrine-loaded ethosomes at a single dose of 0.45 mg/rat and intravenous administration of drug solution at a single dose of 0.45 mg/rat and 30 mg/kg were presented in Figure 10. Tetrandrine could be detected in rat plasma till up to 12 h after intravenous administration at dose of 30 mg/kg, and the tetrandrine concentration in rat plasma at each interval was comparative to that reported previously [39], whereas tetrandrine could not be detected at dose of 2.5 mg/kg, which was equivalent to 0.45 mg/rat of topical formulation, after intravenous administration. Noteworthy, neither could be detected for topical formulation due to the concentration of tetrandrine in plasma was much lower than the limit of detection. This suggested that the systemic toxicity and side effects of the topical formulation of tetrandrine would be neglected.

## 4. Discussion

Transdermal and topical drug delivery has been proposed as the most feasible alternative to parenteral injections due to the avoidance of first-pass metabolism, lower fluctuations in plasma drug levels, targeting of the active ingredient for a local effect, and good patient compliance [40]. However, the barrier nature of skin makes it difficult for most drugs to penetrate into and permeate through it [41]. The improvement of transdermal and topical bioavailability for drugs generally depends on the enhancer or carrier. Topical delivery of drugs by lipid vesicles has evoked a considerable interest. Ethosomes, an interesting and innovative vesicular system, have been used for transdermal drug delivery in recent years because they can enable a drug to pass through the skin and increase the accumulation of drug in the skin [42]. Herein the ethosomes and conventional liposomes were developed and examined for their ability to improve the topical absorption of tetrandrine through dermal delivery, and the relation of the formulations to the pharmacological activity of tetrandrine loaded in the formulations was also assessed.

It is well known that many factors govern the delivery of drugs into the skin from topically applied formulation, wherein vesicular size and EE are the key parameters, which play a decisive role in providing enhanced flux. The vesicles smaller than 300 nm are able to deliver their contents to some extent into the deeper layers of the skin [43]. In the case of our experiment, ethosomes and liposomes had average diameters of smaller than 100 nm; this nanoranged size might present an ample potential for delivery through the skin. In addition, the average diameter of tetrandrine-loaded ethosomes (78.03 ± 0.24 nm) was extremely significantly less than that of tetrandrine-loaded liposomes (99.86 ± 0.48 nm) (*P* < 0.001). This obvious reduction in vesicle size was also reported in previous studies [24] and suggested that it might be attributed to the incorporation of high ethanol concentration. Probably, ethanol causes a modification of the net charge of the system and confers it some degree of steric stabilization that may finally lead to a decrease in the mean particle size [43]. Moreover, the EE of ethosomes (52.87 ± 3.8%) was much lower than that of liposomes (98.8 ± 0.01%), which might be attributed to the fact that the intercalation of ethanol into the PC polar head group environment results in an increase in the membrane fluidity and permeability [24, 28, 44].

Regardless of whether transdermal absorption has a systemic action or a local effect, drugs should pass through the stratum corneum barrier. The ability of different formulations to deliver tetrandrine into and across the rat skin was therefore investigated using Franz vertical diffusion cells. Unlike conventional liposomes, ethosomes were shown to permeate through the stratum corneum barrier and possessed significantly higher transdermal flux in comparison with liposomes (Figures 4 and 6). In other words, the delivery of tetrandrine from ethosomes through the skin was 2.1-fold higher than that from liposomes. The difference in the content of tetrandrine deposited in rat skin at the end of the experiment was also highly significantly greater (*P* < 0.01, 1.7-fold) when delivered from ethosomes compared with liposomes (Figure 5). However, no significant difference between liposomes and solution was observed in terms of the delivery of tetrandrine through the skin (*P* > 0.05) (Figure 4), but extremely significant difference in the content of tetrandrine deposited in rat skin at the end of the experiment was also observed (*P* < 0.001) (Figure 5). These indicated that ethosomes exhibited favorable and enhanced penetration and permeation behavior as compared with liposomes, and liposomes could not provide the required enhancing penetration effect as compared with solution. This phenomenon was confirmed by CLSM observation, and was also found in previous observations of indinavir [45] and 5-aminolevulinic acid [46] ethosomes, which produced significant accumulation of these drugs in the skin. However, the exact mechanism for better permeation into deeper skin layers for ethosomes is still not clear. Some kind of synergistic effects of combination of ethanol, vesicles, and skin, but rather ethanol worked alone have been suggested to be responsible for deeper distribution and penetration in the skin lipid bilayers [24, 28, 44, 47]. In other words, this is owing to the ethanol effect, wherein ethanol interacts with lipid molecules in the polar head group region, leading to a reduction in the transition temperature of stratum corneum lipids, hence increasing their fluidity and decreasing the density of the lipid multilayer [48]. This is followed by the “ethosomes effect”, which includes lipid penetration and permeation by the opening of new pathways due to the malleability and fusion of ethosomes with skin lipids, resulting in the enhanced delivery of the drug in terms of depth and quantity as shown in Figure 6. *In vitro* permeation results were further confirmed by CLSM observation (Figure 6), which showed that liposomes were not able to deeply penetrate skin but remain in the upper layers of the stratum corneum. These were in agreement with the previous reports [24, 44]. On the other hand, our findings of the EE of ethosomes almost being half of liposomes, the significantly higher steady state flux, and the deposition in the skin for ethosomes suggested that the permeation enhancement effect of ethosomes into and across the skin might be effective for both entrapped and nonentrapped drugs [33], which was not corresponded with the previous report [48].

To evaluate a possible correlation between *in vitro* and *in vivo* behaviors, the antiarthritic efficacy of different tetrandrine formulations was evaluated. FCA-induced arthritis in rats has been widely used as an animal model to determine* in vivo* efficacy of anti-inflammation. Injection of FCA into rats resulted in some pathological features such as lassitude, activity lessening, response torpor, forced foot, arthritis nodule in trail, and nasal part tumefaction [49]. In current study, the change in paw edema volume or swelling degree was chosen as an objective parameter to assess the antiarthritic efficacy of the drug. The permeation-enhancing effect of ethosomes and liposomes on tetrandrine was evaluated by topically administering these formulations into FCA-induced rats and assessing antiarthritic response. No significant difference (*P* > 0.05) in the antiarthritic efficacy between liposomes and solution was observed for both primary and secondary adjuvant arthritis in rats (Figures 8 and 9 and Table 2), indicating that liposomes could not enhance the permeation of tetrandrine compared with solution. This relationship was closely corresponded to their trend in skin permeation and CLSM observation. Although the deposition of tetrandrine in the skin from liposomes was extremely significantly greater compared with solution, liposomes might remain in upper layers of the stratum corneum due to the fact that they do not deeply penetrate skin [24, 44], and thereby the treatment effect of tetrandrine loaded in liposomes could not be played on the induced edema. On the contrary, there was a significant difference in the antiarthritic efficacy (*P* < 0.01 for primary adjuvant arthritis, and *P* < 0.01 at day 10 and 15, *P* < 0.05 at day 13, and *P* < 0.001 at day 18 for secondary adjuvant arthritis) between ethosomes and liposomes (Figures 8 and 9 and Table 2), which was also consistent with the result of *ex vivo* skin permeation and deposition. These findings were in good accordance with the previous observations of ammonium glycyrrhizinate [50] and econazole nitrate [51] ethosomes, which were able to significantly enhance the pharmacodynamic effect of these drugs compared with liposomes and the ethanolic or aqueous solutions of these drugs. Based on these results, we considered that this seemed to be attributed to the increasing permeability of the skin to the drug by ethosomes (Figures 4–6).

On the other hand, although there was no significant difference in swelling degree between the market dexamethasone ointment (positive control) and tetrandrine-loaded ethosomes for both primary adjuvant arthritis and secondary adjuvant arthritis (Figure 7), the inhibition rates of paw oedema in rats for tetrandrine-loaded ethosomes were higher for primary adjuvant arthritis, whereas the inhibition rates for dexamethasone ointment were higher for secondary adjuvant arthritis (Table 2). These suggested that tetrandrine-loaded ethosomes appeared to be more applicable in the treatment of primary adjuvant arthritis, whereas dexamethasone ointment might be more suitable for the treatment of secondary adjuvant arthritis.

Results of the drug levels in rat plasma showed that when tetrandrine-loaded ethosomes were topically administered in rats the drug level was too low to be detected in rat plasma. By providing fewer delivery of tetrandrine into the bloodstream, topical administration might offer favorable efficacy with reduced side effects, thus leading to improved patient compliance. In conclusion, ethosomes were demonstrated to be promising carriers for improving topical delivery of tetrandrine via skin. Nevertheless, more detailed studies on tetrandrine-loaded ethosomes need to be further conducted such as physical stability and skin irritation.

## Figures and Tables

**Table 1:** The p*K*_*a*_ values of tetrandrine in different concentrations of hydroethanolic solutions.

Concentration of tetrandrine (mmol/L)	% ethanol (v/v)			
	40	50	60	0
1.50	7.08	7.05	7.06	7.11
1.00	6.46	7.23	6.28	7.12
0.50	6.43	6.73	6.18	7.08

**Table 2:** The inhibition rate (%) of paw oedema in rats at different time for different tetrandrine formulations and market dexamethasone ointment (positive control).

Time	Ethosomes			Positive control	Liposomes	Solution
	High dose	Medium dose	Low dose			
		Primary adjuvant arthritis				
18 h	36.80	13.07	1.85	16.51	1.32	1.60
66 h	46.48	16.97	7.10	38.77	7.79	8.63

		Secondary adjuvant arthritis				
10 d	66.01	34.62	44.72	79.07	43.31	30.70
13 d	62.64	35.37	44.00	78.81	42.76	14.96
15 d	67.60	40.36	41.90	78.99	42.29	14.59
18 d	70.95	46.33	41.13	82.72	53.98	42.66
